# Nanocomposite of Ag-Doped ZnO and AgO Nanocrystals as a Preventive Measure to Control Biofilm Formation in Eggshell and *Salmonella* spp. Entry Into Eggs

**DOI:** 10.3389/fmicb.2019.00217

**Published:** 2019-02-19

**Authors:** Belchiolina Beatriz Fonseca, Paula Luiza Alves Pereira Andrada Silva, Anielle Christine Almeida Silva, Noelio Oliveira Dantas, Aline Teodoro de Paula, Otavio Cintra Lemos Olivieri, Marcelo Emilio Beletti, Daise Aparecida Rossi, Luiz Ricardo Goulart

**Affiliations:** ^1^School of Veterinary Medicine, Federal University of Uberlândia, Uberlândia, Brazil; ^2^Physics Institute, Federal University of Alagoas, Maceió, Brazil; ^3^Institute of Biomedical Sciences, Federal University of Uberlândia, Uberlândia, Brazil; ^4^Institute of Biotechnology, Federal University of Uberlândia, Uberlândia, Brazil

**Keywords:** disinfection, pore, preventive, nanocrystal, bacterium

## Abstract

*Salmonella* spp. is an important foodborne agent of salmonellosis, whose sources in humans often include products of avian origin. The control of this bacterium is difficult especially when *Salmonella* spp. is organized into biofilms. We hypothesized that the novel nanocomposites of ZnO nanocrystals doped with silver (Ag) and silver oxide (AgO) nanocrystals (ZnO:Ag-AgO) synthesized by the coprecipitation method could control or prevent the formation of *Salmonella* Enteritidis (SE) and *Salmonella* Heidelberg (SH) biofilm and its entry into turkey eggs. The diffraction characteristics of ZnO and AgO showed sizes of 28 and 30 nm, respectively. The Zn to Ag substitution into the ZnO crystalline structure was evidenced by the ionic radius of Ag+2 (1.26 Å), which is greater than Zn+2 (0.74 Å). For the SE analyses post-biofilm formation, the ZnO:Ag-AgO was not able to eliminate the biofilm, but the bacterial load was lower than that of the control group. Additionally, SE was able to infiltrate into the eggs and was found in both albumen and yolk. For the SH analyses applied onto the eggshells before biofilm formation, the ZnO:Ag-AgO treatment prevented biofilm formation, and although the bacterium infiltration into the eggs was observed in all treated groups, it was significantly smaller in ZnO:Ag-AgO pre-treated eggs, and SH could not reach the yolk. There was no difference in pore size between groups; therefore, the inhibition of biofilm formation and the prevention of bacterium entry into the egg were attributable to the use of ZnO:Ag-AgO, which was not influenced by the egg structure. Although the amount of Ag and Zn in the shell of the ZnO:Ag-AgO group was greater in relation to the control, this difference was not detected in the other egg components. In the search for new measures that are effective, safe and viable for controlling microorganisms in poultry farming, the application of a nanocomposite of Ag-doped ZnO and AgO nanocrystals appears as an alternative of great potential to prevent *Salmonella* sp biofilms in eggshells and other surfaces.

## Graphical Abstract

Preventive treatment with ZnO:Ag-AgO novel nanoscomposite nanocrystals prevented the SH biofilms formation and it was more efficient than peracetic acid to prevent the entry of bacteria into the eggs. The previous treatment with ZnO:Ag-AgO nanocomposite is a viable alternative to avoid the formation of biofilms and to diminish the bacterial load.

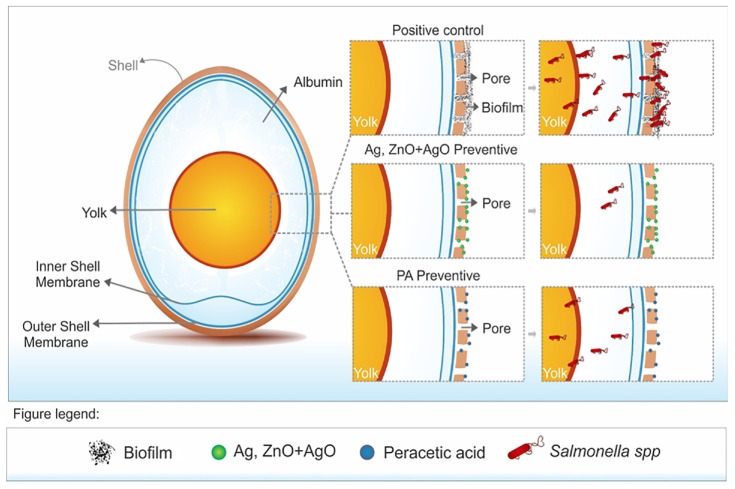

## Introduction

The control of microorganisms is a primordial action for poultry production due to the potential risk of transmission of pathogenic organisms to humans and other animals throughout the entire food chain. Among the most important microorganisms, *Salmonella* spp. may be considered one of the most important foodborne agents in the European Union and the United States (Center for Disease Control CDC, 2016; EFSA, 2017) and may cause significant damage to the poultry industry as well as to public health. Importantly, products of avian origin represent 47% of salmonellosis sources in humans (Center for Disease Control CDC, 2016).

Control of the different serotypes of *Salmonella* spp. is required throughout the entire poultry production cycle. Embryonated eggs, equipment, facilities, vehicles and other materials involved in the process must undergo an efficient disinfection to control these microorganisms. Historically, formaldehyde has been chosen as one the main disinfectant agent of eggs, however, its use has been restricted (BRASIL, 2008), openning space to other disinfectants, such as the peracetic acid (PA).

Current research has sought alternatives for the effective biological control of avian products that are not only harmless to humans, other animals and the environment, but also easily applicable at a feasible cost. This scenario has introduced nanoparticles as a potential alternative biocidal agent, due to their ability to achieve intimate interactions with bacterial surfaces conferred by their small size, a property that results in a high surface-to-volume ratio, thus providing a high biocidal power (Allaker, 2010).

Zinc oxide (ZnO) nanocrystals (NCs) are a biocompatible materials, according to the U.S. Food and Drug Administration (FDA); therefore, in this work we investigated this nanocrystal doped with silver (Ag) and with silver oxide (AgO) nanocrystals. ZnO nanocrystals (NCs) exhibit many important characteristics, such as high catalytic activity, chemical and physical stability, as well as ultraviolet (UV) absorption (Tankhiwale and Bajpai, 2012). The doping technique is employed to produce defects aiming to increase the catalytic activity in ZnO NCs, and consequently, the ability to induce oxidative stress in bacteria, which is one of the main mechanisms of bactericidal action (Pati et al., 2014). The catalytic activity is a property that induces the generation of reactive oxygen species (ROS), leading to oxidative stress and bacterial death (Espitia et al., 2012). The mixing of NCs into nanocomposites (NC) can potentiate a certain physical property or the presence of two interesting physical properties. Thus, in the present work, we investigated the synergism of Ag-doped ZnO with AgO NCs in the bactericidal action.

In order to ensure survival in harsh environments, *Salmonella* spp., as well as other microorganisms, develop adaptation mechanisms, such as biofilm formation (Steenackers et al., 2012). Biofilm is the aggregation of sessile microorganisms on a surface, followed by self-production of extracellular matrix, composed of protein and exopolysaccharides (Donlan and Costerton, 2002; Martínez and Vadyvaloo, 2014). This mechanism confers to the bacteria capacities of persistence and survival in adverse environments, and especially, resistance to the action of biocides, since this physical barrier hinders the action of the disinfectant agent (Nikolaev and Plakunov, 2007; Tomihama et al., 2007; Flemming and Wingender, 2010). The formation of biofilms is a great concern given their ability to remain in environments, resisting sanitizers and antimicrobials, and give rise to a constant source of microorganisms, favoring pathogen propagation in production chains and, consequently, in foods (Schlisselberg and Yaron, 2013).

Nanocomposites consist of a mixture of different nanocrystals that may synergize and potentiate certain physical properties due to the combination of two interesting and complementary physical properties. Zinc oxide (ZnO) is generally recognized as safe (GRAS) by the U.S. Food and Drug Administration and may be used as food additives due to its antimicrobial properties. ZnO nanoparticles present pronounced antimicrobial activity due to their small size and high surface-to-volume ratio of nanoparticles, showing selective toxicity to bacteria and minimal effects on human cells (Reddy et al., 2007), most likely due to disruption of the bacterial membrane and oxidative stress (Xie et al., 2011; Espitia et al., 2012; Pati et al., 2014). Another important nanoparticle with significant antimicrobial effect is Silver Oxide (AgO). AgO has been used as cleaning and preservative agents due to its high catalytic activity and selectivity as catalyst in organic reactions, however, the antimicrobial activity of AgO nanoparticles against bacteria depends on their shape (Wang et al., 2010).

Considering the necessity and importance of controlling microorganisms, especially *Salmonella* spp. in relation to avian products, the present study aimed to verify the synergistic action of nanocomposites composed of Ag-doped ZnO containing AgO NCs (ZnO:Ag-AgO) on the control and prevention of *Salmonella* Enteritidis (SE) and *Salmonella* Heidelberg (SH) in biofilm formation in turkey embryonated eggshells, which present a rough surface with many pores that allow bacterial contamination during cleaning prior to consumption. Herein, we apply for the first time a nanocomposite as an antimicrobial agent on eggshells to prevent biofilm formation, which may have an important impact on the poultry industry.

## Materials and Methods

### Synthesis and Characterization of the NCs of ZnO:Ag-AgO

The synthesis of the ZnO:Ag-AgO nanocrystals was performed at the “Laboratory of New Insulating and Semiconductor Materials (LNMIS)” at the Institute of Physics of the Federal University of Uberlândia. ZnO:Ag-AgO NCs were synthesized by the coprecipitation method, which is under a patent application (BR 10 2018 007714 7). The physical properties of the NCs were investigated by X-Ray Diffraction (XRD) and electronic scanning microscopy (SEM). XRD patterns were measured by a Shimadzu XRD-6000 diffractometer with a Cu-target radiation (λ = 0.154 nm). The SEM images and EDS results were obtained by scanning electron microscopy (SEM; Carl Zeiss SMT Ltd., EVO MA 15). All characterizations were performed at room temperature.

### Evaluation of the Concentration of Ag-Doping for Inhibition of *Salmonella* spp.

The biological studies and eggs’ incubation were performed, respectively, in the Laboratory of Molecular Epidemiology and Laboratory of Egg Incubation of the School of Veterinary Medicine at the Federal University of Uberlândia. To determine the activity of ZnO:Ag-AgO, we have used antibiograms according to Bauer et al. (1966), which consisted of inoculating a bacterial suspension (SE and SH) on Mueller Hinton agar plates, followed by the application of filter paper disks impregnated with 1.4 mg/mL of nanoparticles under three different dosages of Ag-doping (5, 9, and 11%). Sulfonamide disks (300 μg; LABORCLIN^®^) were used as a control. We incubated the plates for 20 h at 37°C. After incubation, the growth or inhibition pattern was analyzed around each disk, and the halos were measured for the determination of the inhibition spectrum.

### Efficiency of ZnO:Ag-AgO NCs in the Elimination of SE and SH in Biofilm Formation in Eggshell and Entry Into Eggs

The eggs originated from a 35-week-old turkey breeder from an industrial poultry business. The SE strain used in the study was provided by the Instituto Oswaldo Cruz Foundation (FIOCRUZ) and the SH strain was isolated in our laboratory from broilers, where they were characterized and typed genetically.

The efficiency of the ZnO:Ag-AgO nanocomposite in the control of SE and SH biofilms was evaluated in eggs. As to SE, we divided 64 eggs into four groups, each group consisting of 16 eggs and each sample unit consisting of two eggs. For biofilm formation, three groups were submerged, separately two by two, in a 100 mL suspension of TSB containing 10^5^ CFU/mL of SE, for a period of 24 h at 25°C. In the fourth group (negative control - NC), the eggs were also submerged for 24 h, but in sterile TSB. After this period (24 h) the eggs were washed three times in ultrapure water to remove free bacteria from the biofilms. The eggs were dried, after which the disinfectant treatments were applied: (i) ZnO:Ag-AgO: Spraying 1.4 mg of ZnO:Ag-AgO nanocomposite diluted in water per egg during 30 min; (ii) PA: Immersion in peracetic acid 2.5% (25,000 ppm) for 15 s; (iii) PC (positive control): Spray of ultrapure water, previously in contact with TSB containing Bacteria; and (iv) NC (negative control): Spray of ultrapure water, previously in contact with sterile TSB. After drying, the eggs were opened in laminar flow, separated aseptically into shell, albumen and yolk, and collected.

### Bacterial Identification and Counting for SH and SE

We evaluated a total of 10 g of each sample unit both qualitatively and quantitatively. In the quantitative evaluation of the shell, a total of 10 g of the sample was added to 90 mL of 0.9% NaCl solution, thoroughly homogenized and submitted to serial dilutions for subsequent plating on TSA agar surface incubated at 37°C for 30 h for SE counting. At the same time, the presence and absence of SE in the shell, albumen and yolk were evaluated. A 10-gram sample of shell and 1 mL of albumen and yolk were added to 90 mL and 9 mL of peptone water (Isofar^®^), respectively, and separately. These samples were incubated for 24 h at 37°C; then a 1mL aliquot of this was inoculated into Rappaport (Oxoid^®^) culture medium and 1mL in Tetrathionate (Merck^®^) culture medium. After 24-h growth, both were depleted in XLD. Colonies with morphological characteristics of Salmonella were selected; three to five CFU per plate were submitted to a conventional PCR reaction for specie confirmation. As the eggs were only disinfected and not sterilized, another microarray PCR assay was performed to confirm serotypes. The multiplex ligation detection reaction (LDR) generated collections of DNA molecules. Such DNA molecules were subsequently amplified by means of a single pair of amplimers through a PCR. The PCR products were then sorted by hybridization by a low-density DNA microarray. Positive hybridization was detected using a biotin label incorporated in one of the PCR primers. Tubes were then inserted in the single-channel ATR03 array tube reader upon completion of the detection reaction, and images were acquired and interpreted via the software supplied by the manufacturer (Check-Points, Wageningen, Netherlands).

### Efficiency of NCs of ZnO:Ag-AgO in Prevention of SH Biofilm Formation in Eggshell and Entry Into Eggs

The results of the SE experiments led us to make some modifications to the experiments on SH. To evaluate the efficiency of the nanoparticle in the control SH biofilms, the same methodology for bacteria inoculation and eggs analysis described above were used. However, two more groups were added: (i) ZnO:Ag-AgO pre (preventive treatment with ZnO:Ag-AgO): ZnO:Ag-AgO was applied twice, being approximately 1.4 mg/mL per egg in each application. A spray was performed 24 h before contact with the SH and another treatment 30 min after the biofilm formation and the removal of the free forms of the bacterium; (ii) ZnO:Ag-AgO post (treatment after biofilm formation with nanocomposite of ZnO:Ag-AgO): ZnO:Ag-AgO spray (1.4 mg per egg) was applied for 30 min after biofilm formation and removal of free forms of the bacteria; (iii) PA pre (preventive treatment) with peracetic acid: identical to NP pretreatment but using 25,000ppm of peracetic acid per egg; (vi) PA post (treatment after biofilm formation with peracetic acid): identical to treatment NP post but using 25,000ppm of peracetic acid per egg; (v) PC: Ultrapure water spray 24 h after biofilm formation; (vi) NC: Ultrapure water spray 24 h after immersion in sterile TSB. The same laboratorial experiments carried out for SE identification and quantification were conducted for SH.

### Evaluation of Eggshell Pore Size

Considering the possibility that shell pore diameter may influence the nanocomposite treatments applied before and after SH contamination, we used confocal Raman microscopy. Images obtained from the equatorial region of the eggshells were evaluated by the program Gwyddion to obtain the figures with measurements of shell roughness height and pore diameter.

### Scanning Electron Microscopy

The SH biofilm was evaluated by SEM, following the protocol described by Brown et al. (2014) with modifications. Biofilms formed in eggshells were fixed with 2.5% glutaraldehyde and 2.5% paraformaldehyde in 0.1 M buffer PBS (pH 7.4) overnight at 4°C. The fixed material was removed, and the samples were washed three times with PBS buffer. The eggshell was fixed again with 1% osmium tetroxide for 2 h and washed three times with PBS buffer. The eggshell was then dehydrated several times by using ethanol solutions at the following concentrations: 30, 40, 50, 60, 70, 80, and 90% and then three times at 100% for 20 min at each concentration. The samples were dried in Critical Drying Point (CPD; CPD 030, Baltec, Germany) using liquid carbon dioxide as the transition fluid. The samples were coated with a 20-nanometer-thick layer of gold (SCD 050, Baltec, Germany) and visualized on SEM (VP Zeiss Supra 55 SEM FEG operating at 5 kV).

### Levels of the Ag and Zn in Egg Components

A total of three samples of albumen, yolk and eggshell from the NPpre and C (control -without nanoparticle) groups were analyzed to identify the presence of Ag and Zn, and thus to understand the invasiveness level of the nanoparticles in the eggs. A total of 1 g of each sample was muffled at 600**°**C for 3 h, then cooled in a desiccator to obtain the dry matter. The samples were then ground into a homogeneous powder form. Then 0.1 g of each sample was weighed and added to 6 ml of a nitro-perchloric solution, with the nitric and perchloric acid at the ratio of 8:1. During the digestion of the samples, there was a controlled variation of temperature: 15 min at 50**°**C, 15 min at 75**°**C, 30 min at 120**°**C, 40 min at 160**°**C and finally at 60 min at 210**°**C, to finally obtain approximately 5 ml of translucent extract. After cooling, 50 ml of water was added at 60°C and homogenized. The samples were filtered and submitted to Inductively Coupled Plasma Optical Emission Spectrometry (ICP-OES) and then the concentrations of Ag and Zn were quantified.

### Statistical Analysis

The analyses were performed with the aid of the software GraphPad Prism, version 7.0. The quantitative biofilm formation tests performed on the eggshells were analyzed by the Kruskal–Wallis test followed by Dunn’s multiple comparisons test. For the difference between percentages, the Chi-square test followed by the Fisher–Irwin tests of two-by-two was used. ANOVA and the Tukey test were employed to analyze the shell roughness height and the Ag and Zn amounts we used. The confidence level was 95% for all reports.

## Results

### Characterization of ZnO:Ag-AgO Nanocomposites

**FIGURE 1:**
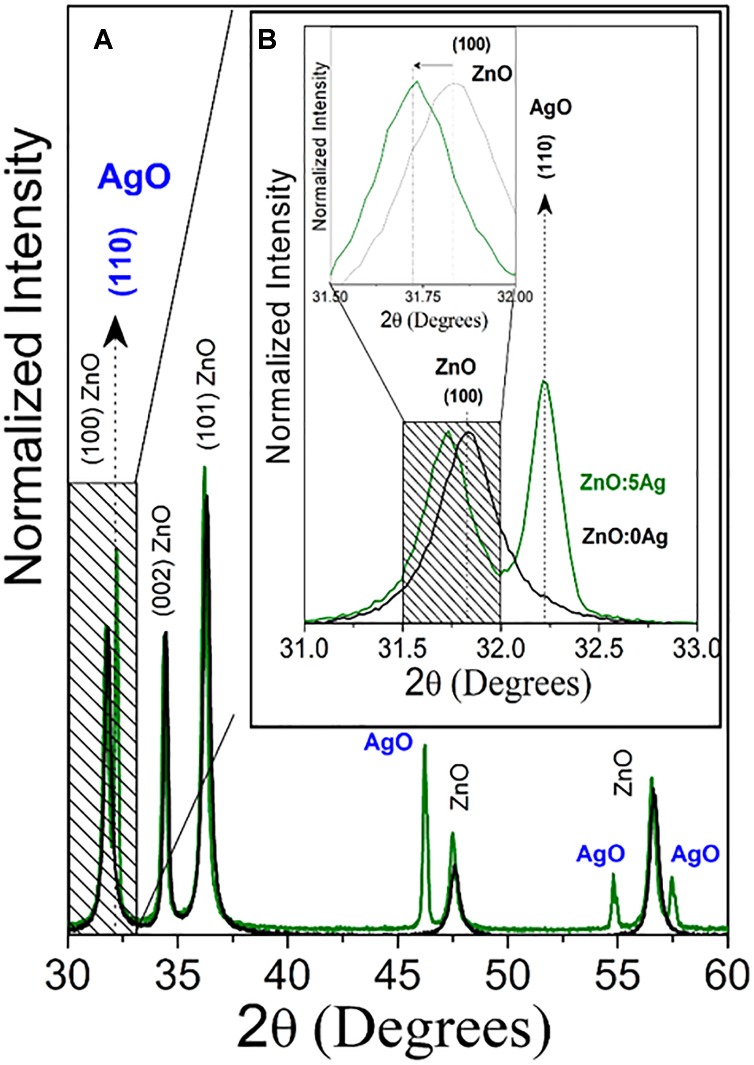
**(A)** X-ray patterns of ZnO nanocrystals (black line) and nanocomposite (ZnO:5Ag; green line). **(B)** Amplification to investigate the alterations of the ZnO main peak due to the incorporation of Ag.

The structural and morphological properties of the ZnO NCs and NC were investigated by XRD and electronic SEM, respectively. Figure 1 shows the XRD characteristic diffraction patterns of wurtzite ZnO nanocrystals (black line) and nanocomposite (green line). An amplification was performed to investigate the alterations of the ZnO main peak due to the incorporation of Ag. (Figure 1B inset). Figure 1B shows a shift to smaller angles relative to the ZnO peak (100), and this result confirms the substitutional incorporation of silver in place of zinc into the ZnO crystalline structure, since Ag^+2^ has an ionic radius (1.26 Å) greater than Zn^+2^ (0.74 Å) and the peak characteristic diffraction of AgO nanocrystals. The AgO NCs percentage formed was calculated by XRD patterns and obtained 49.36%. These results confirmed the formation of nanocomposite of Ag-doped ZnO with AgO nanocrystals.

**FIGURE 2:**
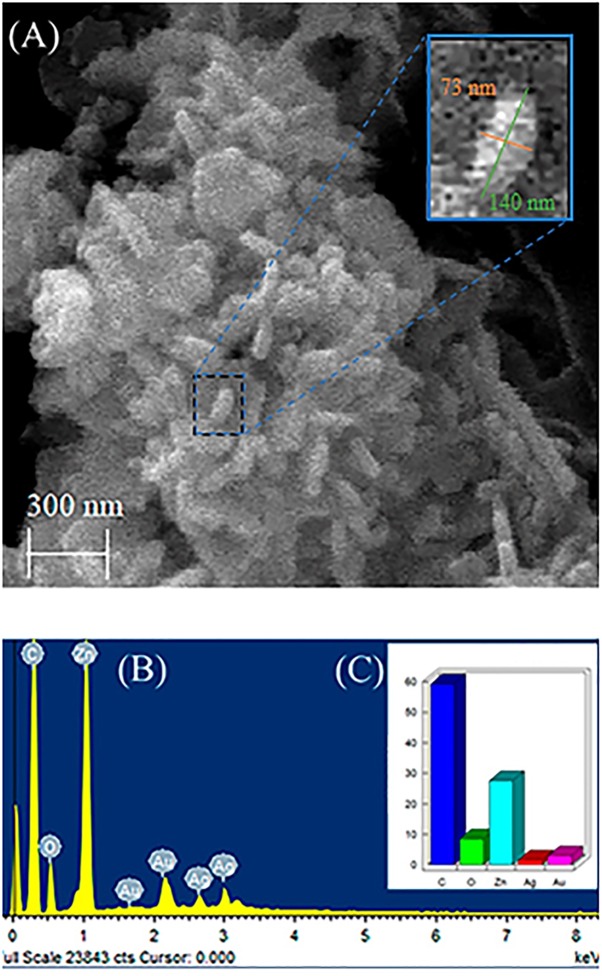
**(A)** Scanning microscopy image of nanocomposite (ZnO:5Ag), **(B)** EDS results, and **(C)** Quantitative elements in weight%.

Figure 2A shows SEM image of nanocomposite (ZnO:5Ag) confirming the rod-shaped Ag-doped ZnO nanocrystals with diameter of 73 nm and length of 140 nm, as well as the morphology of AgO NCs, which is a mixture of plates with a sphere. Figure 2B shows EDS results that confirmed the presence of Ag, Zn and O elements, which are the constituents of Ag-doped ZnO and AgO NCs in excellent agreement with XRD results (Figure 1). Figure 2C shows the quantitative elements in weight %. The elements C and Au consist of the substrate and metallization, respectively.

The antibiogram results showed that 5% Ag-doping was sufficient to inhibit SE and SH. Therefore, we have used this percentage of doping.

### ZnO:Ag-AgO NCs Were Not Able to Decrease the Positivity of Biofilm Formation in Eggs but Reduced the Contaminant Load of SE

**FIGURE 3:**
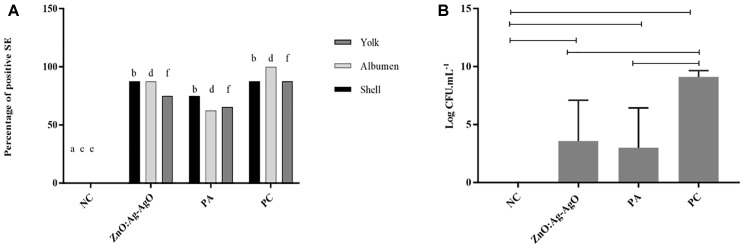
Evaluation of the efficiency of ZnO:Ag-AgO in the manage the already formed SE biofilm. **(A)** Percentage of shell, albumen and yolk positive for SE submitted to different types of disinfection. Different letters represent statistical differences in the presence of SE in the shell (a,b), albumen (c,d) or yolk (e,f) of the eggs. NC: negative control; PA: Peracetic acid; PC: Positive control. **(B)** Bacterial load on SE biofilms formed in turkey eggs subjected to different types of disinfection. ZnO:Ag-AgO and PA caused a significant drop in the contaminant load on SE biofilms formed in turkey eggs. NC: negative control; PA: Peracetic acid; PC: Positive control.

In the SE analyses, the biofilm was formed in all groups that were exposed to the bacterium. *Salmonella* was able to advance into the egg, being isolated in albumen and yolk. Figure 3A shows that there was no statistical difference between the percentages of contaminated eggs in eggshell, albumen or yolk among the ZnO:Ag-AgO, PA and PC groups. Nanocomposite of ZnO:Ag-AgO and PA were not efficient at decreasing the percentage of eggs positive for the presence of biofilm on their shells and the passage of the SE into the egg (Figure 3A). However, nanocomposite of ZnO:Ag-AgO and PA caused a significant drop in the contaminant load (Figure 3B). Compared to the PC group, the reduction caused in ZnO:Ag-AgO was 5.538 Log CFU.mL-1, whereas in PA it was 6.092 Log CFU.mL-1 (Figure 3B).

### ZnO:Ag-AgO NCs Prevent SH Biofilm Formation in Eggshell and Decreased the Entrance of the Bacterium Into the Egg but Do Not Eliminate Previously Formed Biofilm

**FIGURE 4:**
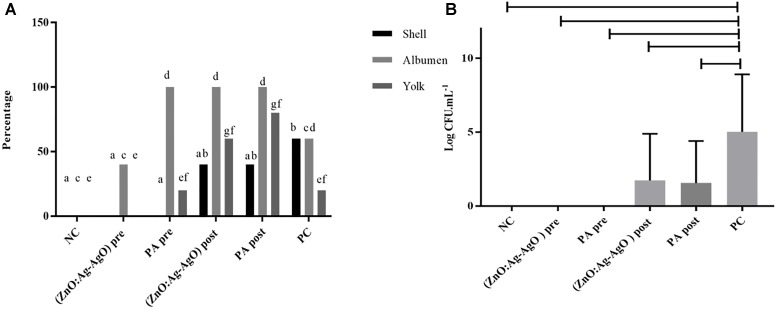
Evaluation of the efficiency of ZnO:Ag-AgO in the manage the already formed SH and in the prevention of SH biofilm formation. **(A)** Percentage shell, albumen and yolk positive for SH submitted to different types of disinfection. Different letters represent statistical differences for the presence of SH in the shell (a,b), albumen (c,d) or yolk (e,f) of the eggs. (ZnO:Ag-AgO) pre and PA pre prevented the biofilm formation. (ZnO:Ag-AgO) pre: preventive treatment with nanocomposite of ZnO:Ag-AgO; PA pre: preventive treatment with peracetic acid; NC: negative control; (ZnO:Ag-AgO) post, treatment after biofilm formation with nanocomposite of ZnO:Ag-AgO; PA post: treatment after biofilm formation with peracetic acid; PC: Positive control. **(B)** Bacterial load on SH biofilms formed in eggshell subjected to different types of disinfection. (ZnO:Ag-AgO) pos and PA post caused a significant drop in the contaminant load on eggshell SH biofilms. (ZnO:Ag-AgO) pre and PA pre prevented the biofilm formation. (ZnO:Ag-AgO) pre: preventive treatment with nanocomposite of ZnO:Ag-AgO; PA pre: preventive treatment with peracetic acid; NC: negative control; (ZnO:Ag-AgO) post: treatment after biofilm formation with nanocomposite of ZnO:Ag-AgO; PA post: treatment after biofilm formation with peracetic acid; PC: Positive control.

There was no biofilm formation in eggshell in both preventive treatments by means of ZnO:Ag-AgO and PA (denominated (ZnO:Ag-AgO) pre and PA pre, respectively (Figure 4). The passage of the bacterium into the eggs was noted in all treated groups, being smaller in ZnO:Ag-AgO pre (Figure 4A). In this group, the percentage of SH-positive albumin was lower than in the other treated groups and the bacteria did not reach the yolk (Figure 4A). Evaluating the efficiency of the SH biofilm inhibition of on eggshell, the nanocomposite of ZnO:Ag-AgO and peracetic acid post treatment caused a significant drop in the contaminant load (Figure 4B).

**FIGURE 5:**
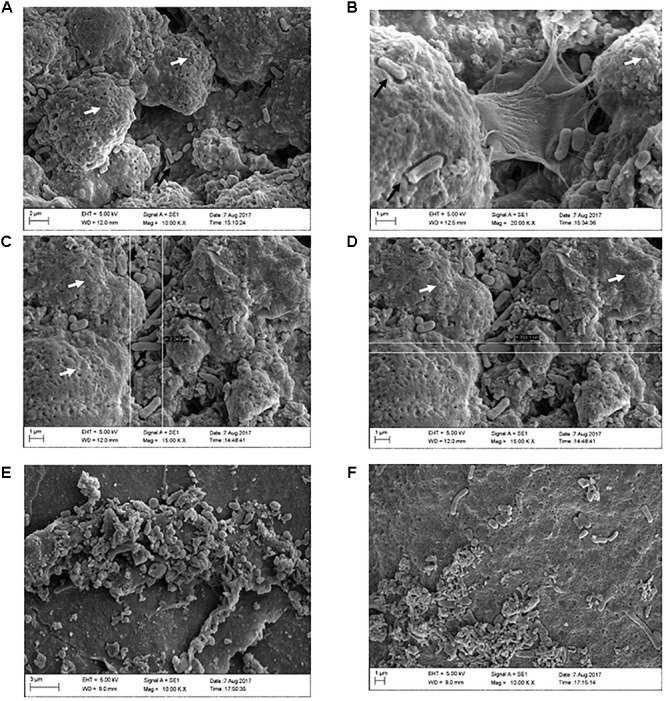
Formation of SH biofilms on an eggshell in PC, (ZnO:Ag-AgO) pre and NC groups. **(A)** PC – Typical SE biofilm matrix formation (as seen on the white arrows), with invasion of bacteria as indicated by the black arrows. **(B)** PC – Biofilm of SH in stage of expansion, with visualization of the bacteria releasing the matrix. **(C)** PC – Biofilm of SH and presence of free bacteria on the surface. Length measurement (2,245 μm). **(D)** PC – Biofilm of SH and presence of free bacteria on the surface. Width measurement (703.1 nm). **(E)** (ZnO:Ag-AgO) pre – Structure similar to the SH biofilm matrix, however, it is unconfigured and bacteria are visualized in different formats. **(F)** NC – Group of cells in different formats verified near the unconfigured matrix.

In order to verify whether the use of ZnO:Ag-AgO nanocomposite, either preventively or after the formation of biofilms, was influenced by eggshell pore size, we used Confocal Raman Microscopy (Supplementary Figure S1). As expected, when we measured the size of the SH by SEM (Figure 5B,C), we realized that the eggshell pore size allows passage of SH into the eggs. There was no statistical difference in pore size between groups (Table 1).

**Table 1:** Measurements of pore diameters in eggs submitted or not to different types of disinfection with nanoparticles.

	NC	(ZnO:Ag-AgO) post	(ZnO:Ag-AgO) pre
Pore diameters (μm)	4.032	4.095	4.536


*There was not statistical difference in the eggs pore size.*

Through SEM analyses, we can elucidate the conformation of SH biofilms in eggshells. In the group PC we observed characteristic formation of biofilm as well as free bacteria on the surface of these biofilms (Figure 5A–D). In groups (ZnO:Ag-AgO) pre, AP pre, and NC, we observed a sparsity of matrix that was incompatible with that presented by the group PC. We observed some bacteria in NC but detected no bacteria similar to PC in size and morphology (Figure 5E,F).

Small amounts of both matrix and other microorganisms were expected in the NC since the eggshells were not sterilized. Most of the bacteria found in the NC are probably environmental ones. Furthermore, in the eggshells we detected the presence of organic remnants, such as mucoproteins and other environmental substances, including nest material and suspended feed powder. However, our analysis detected greater differences between negative and positive groups in relation to the matrix formation, shape and bacterial amount. Figure 5 strongly confirmed that the SH biofilm was not formed in the previously treated eggshell with ZnO:Ag-AgO.

### Zn and Ag Ions Have Not Reached Into the Eggs Treated With ZnO:Ag-AgO NCs

As expected, the Ag and Zn, quantified by inductively Coupled Plasma Optical Emission Spectrometry (ICP-OES), showed a large presence of Ag on the shell treated with ZnO:Ag-AgO nanocomposite, but most important, Ag ions have not been detected in the albumen and yolk (Table 2). Zinc was detected in the shell, albumen and yolk samples. Although the amount of Ag and Zn in the shell of the ZnO:Ag-AgO group was statistically greater relative to the control, this difference was not observed in the other egg components (Table 2).

**Table 2:** Concentration of Ag and Zn (ppm) in shell, albumen or yolk of eggs treated with ZnO:Ag-AgO and NC.

	Shell	Shell	Albumen	Albumen	Yolk	Yolk
	NC	ZnO:Ag-AgO	NC	ZnO:Ag-AgO	NC	ZnO:Ag-AgO
**Ag**	0,007a	0,100b	0,003a	0,010a	0.002a	0.001a
**Zn**	0.218a	0.797b	0.265a	0.232a	1.294c	1.295c


*Different letters on the same line for same element means statistical difference.*

## Discussion

In the present work, we have demonstrated that a novel nanocomposite of Ag-doped ZnO and AgO nanocrystals has been successfully used as a preventive measure to control biofilm formation, and SE and SH infiltration into turkey eggs. This new nanocomposite has shown diffraction patterns of both ZnO and AgO, with respective sizes of 28 nm and 30nm. We have also confirmed the Ag doping with substitution of Zn by Ag into the ZnO crystalline structure, by demonstrating that the Ag+2 has an ionic radius (1.26 Å) greater than Zn+2 (0.74 Å). This doping strategy has increased the catalytic activity in ZnO NCs, improving its ability to induce oxidative stress in bacteria (Pati et al., 2014).

*Salmonella* control is a major concern for poultry producers in Brazil since the country is the largest exporter of poultry meat and eggs. The control of SH is quite difficult and highly prevalent in Brazil (Voss-Rech et al., 2015). In poultry products *Salmonella* spp. was the main cause of early warnings in the European Union between January 2017 and May 2018 (RASFF, 2018). It is possible that these bacteria remain in the farm environment as a biofilm form.

We have also used the ZnO:Ag-AgO to control the SE biofilms in eggs and its entry into albumen and yolk, but the nanocomposite was not able to eliminate previously formed biofilms, although has significantly reduced the bacterial load in the egg surface. The presence of *Salmonella* into eggs corroborates previous findings elsewhere (Gustin, 2003), in which *Salmonella* spp. is able to enter the egg contents after 24 h of contact with eggshells. However, we emphasize that the most important finding was the use of ZnO:Ag-AgO before SH biofilm formation, which not only protected from its formation, but also demonstrated that it has effectively blocked the bacterial entry into the albumen and yolk.

Eggshells present numerous pores that are sufficiently large to act as gateways for microorganisms (Cardoso et al., 2001; Silva, 2005). Considering that the shell pore diameter may be involved in the bacterial contamination and translocation into the egg, which may also facilitate the entry of nanocrystals, we have shown for the first time the pore size of turkey eggshells by Confocal Raman, and we also showed that toxic Ag ions did not reach the albumen and yolk, which was also successfully demonstrated by ICP-OES, demonstrating that no ions were internalized. Similar to Ag, higher Zn ion concentration was only observed at the eggshell surface treated with ZnO:Ag-AgO, demonstrating that although the ultra-small size of the ZnO:Ag-AgO NCs would easily allow their entry into the egg, they were not able to translocate to the inner side of eggs. We hypothesize that probably the surface charges of both eggshell and nanocrystals have favored the nanocrystals retention at the shell surface.

It is worth to note that microorganisms are highly resistant to disinfectants when structured as biofilms (Ziech et al., 2016). In general, disinfectants are not capable of eliminating the entire biofilm matrix, requiring further association with a mechanical processes to disturb the matrix structure and to expose the microorganisms to disinfectants (Maukonen et al., 2003; Srey et al., 2013). Although complete elimination has not occurred in the present study, the evaluated disinfectants significantly reduced the microbial load of the biofilm on treated eggs by 5 to 6 logs. Again, the similar performance between the ZnO:Ag-AgO and PA groups as control measures confirmed the potential of the ZnO:Ag-AgO nanocomposite, since the dose of PA used was much higher than the one recommended by the manufacturer. Several studies have shown full or partial efficiency of ZnO or AgO nanoparticles in inhibiting biofilm activity of different microorganisms in polystyrene wells or another material (Sinisa et al., 2015; Singh et al., 2016, 2018; Melo et al., 2017), but none has shown nanocrystals behavior in eggshells and their activity at the surface and within the egg components. Therefore, this is the first time an Ag-doped ZnO-AgO nanocomposite is successfully used as a preventive measure to protect eggs from *Salmonella* spp infection and biofilm formation.

Briefly, in our search for new effective, safe and viable measures to control microorganisms in the poultry farming, we have synthesized a novel nanocomposite that may become an alternative of great potential to effectively prevent *Salmonella* biofilm formation and infection of eggs. Moreover, since eggshells have a rough surface, we believe this in an excellent experimental model for biofilm formation, which reinforces its use on other surfaces. Besides its successful activity against the most important bacterial problem in the poultry industry, it is a harmless substance. Other studies should be performed to ascertain the best administration and control of other microorganisms.

## Author Contributions

BF and LG have developed and planned the project, analyzed and interpreted the data, and wrote the manuscript. AS and ND produced and characterized the nanoparticles. PS, AdP, DR, BF and OO performed microbiology and molecular assays. OO performed the ICP analyses. MB performed and analyzed the biofilm by SEM, and has partially contributed to the manuscript writing. All authors read and approved the final manuscript.

## Conflict of Interest Statement

The authors declare that the research was conducted in the absence of any commercial or financial relationships that could be construed as a potential conflict of interest.

## Supplementary Material

FIGURE S1 Image obtained in Raman confocal microscopy, presence of roughness and pores on turkey eggshells. The arrows in image **A** show the pores of the shell, while **B** depicts the eggshell dimensions in a 3D image.
