# Supplementary figures and images for: Nanocomposite of Ag-Doped ZnO and AgO Nanocrystals as a Preventive Measure to Control Biofilm Formation in Eggshell and Salmonella spp. Entry Into Eggs
Source: Front Microbiol. 2019 Feb 19;10:217. doi: 10.3389/fmicb.2019.00217 (PMC6389690; doi:10.3389/fmicb.2019.00217)

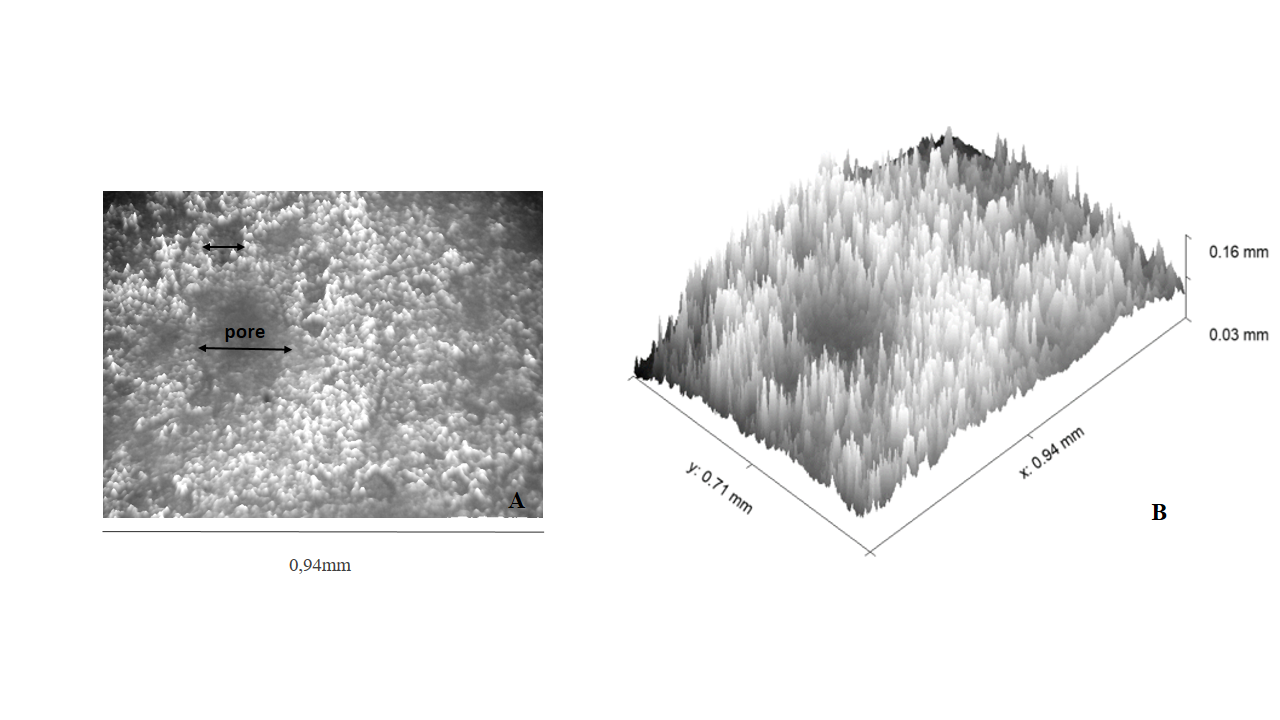

Supplement: FIGURE S1 — Image obtained in Raman confocal microscopy, presence of roughness and pores on turkey eggshells. The arrows in image A show the pores of the shell, while B depicts the eggshell dimensions in a 3D image. [file Image_1.TIF]
